# Hemorrhagic Cystitis due to BK Reactivation in a Young Female Treated for Hodgkin-Disease

**DOI:** 10.1155/2011/592470

**Published:** 2011-07-09

**Authors:** R. Le Calloch, J. C. Ianotto, C. Berthou, A. Tempescul

**Affiliations:** Department of Clinical Hematology, Institute of Cancerology and Hematology, CHU Brest, 2 avenue Foch, 29609 Brest Cedex, France

## Abstract

Hodgkin's lymphoma is a disease with a high rate of curability under classic chemo-radiotherapy regimes. Complications due to chemotherapy could include viral reactivation due to chronic lymphopenia. BK virus (BKV) is a polyoma virus belonging to the Papovaviridae family with antibody seroprevalences in healthy populations varying from 60% to 80%. Initial infections are asymptomatic usually occur in early childhood, after which the viruses remain latent in the kidneys or urothelium. Reactivation of BKV occurs in individuals with severe immunosuppression during HIV infections, transplantation or, exceptionally, after classical chemotherapy. BKV incidence is approximately 0% to 5% in immunocompetent individuals. Reactivation is associated with nephropathy and haemorrhagic cystitis. Herein, we present a case of a haemorrhagic cystitis due to BKV reactivation in a patient with Hodgkin's disease treated with chemotherapy.

Hodgkin's lymphoma is a disease with a high rate of curability with classic chemotherapy [1]. Complications due to chemotherapy could include viral reactivation due to chemotherapy-induced cytopenia [2]. 

BK virus is a polyoma virus from papovaviridae families, finally identified in 1971. Antibody seroprevalence in healthy population varies from 60% to 80%. Initial infections with BKV are thought to be asymptomatic and usually occur in early childhood (BKV), after which the viruses remain latent in the kidneys or urothelium [3, 4]. 

Reactivation BKV infections occur in individuals with severe immunosuppression like HIV infections [5], transplantation [6], or exceptionally after classical chemotherapy. BKV can be detected in 0% to 5% immunocompetent individuals. Reactivation is associated with nephropahty or hemorrhagic cystitis [7]. Currently, no antiviral agent has proven clinical efficacy against BKV.

We present here the case of an hemorrhagic cystitis due to BKV reactivation in a patient treated with chemotherapy for Hodgkin's disease.

A 15-year-young female was admitted to the hospital because of severe hemorrhagic cystitis. The patient was treated for a stage 4 Hodgkin disease, with chemotherapy using COOP then OPPA French protocol. The patient ends the last cure of chemotherapy two weeks before the apparition of the symptoms. Four days before hospitalization, the patient presented moderate fever, 38°C, chills, and very painful cystitis, treated with antibiotics. On admission to the hospital, the patient has macroscopic hemorrhagic cystitis, pain, and pollakiuria. Physical examination revealed marked suprapubic tenderness. Blood count found hemoglobin 11.6 g/dL, leukocyte 13.3 × 10^9^/L, PNN 11.97 × 10^9^/L, lymphocyte 0.27 × 10^9^/L, and platelets 98 × 10^9^/L. Coagulation was normal with INR at 0.94 and TCA at 32/34. C-reactive protein concentration was moderately elevated at 22.2 mg/L. Cytobacteriological examination of urine samples found no bacterial or fungi infection. Abdomen and pelvis ultrasound examination found an enlargement of calyceal cavities at 11 mm, without image of nephrolithiasis, and the diffuse enlargement of bladder associates with images of clot. Hemocultures were negatives. Real-time PCR for adenoviruses was negative. Diagnosis was made by the genomic detection of BK virus in urine with a viral charge at 1.95 mil copies/mL (6.3log). BK viremia was negative. No other viruses (JC, EBV, CMV, HHV6, or HHV8) were detected. 

Because of initial presentation and the recent history of chemotherapy including cyclophosphamide, the first treatment was acrolein inhibitor Uromitexan (Mesna) and morphine, associated to continuous bladder irrigation and Oxybutynin (Ditropan). Acrolein inhibitor treatment was stopped after the detection of BK viruses in urine samples. Continuous bladder irrigation was performed for 10-day time necessary for remission of symptoms. Because of the absence of viremia, no specific antiviral treatment was administrated. The symptoms were spontaneously regressive after 10 days, when the urine sample found BK virus at 35800 copies/mL, allowing the patient to leave the hospital. No other viral charges were performed in the absence of the symptoms.

Hodgkin's lymphoma is a highly curable disease by chemotherapy and radiotherapy, with a majority of patients presenting in early stages of disease [1]. Treatment complications could be early complications like infections, renal failure, hart failure, and so forth, and late complications like secondary acute leukaemia and myelodysplastic syndrome [2]. Early viral complications, about 4%, are mostly herpes virus reactivations with clinical expression of zona, rarely cytomegalovirus pneumonia. 

BK virus is a human polyoma virus, finally identified in 1971, which has been implicated in hemorrhagic cystitis in patients who have undergone allogenic stem cell transplantation [3, 4]. BK virus has also been shown to be the cause of hemorrhagic cystitis, tubulointestinal nephritis in immunodeficient, VIH-positive patients, vasculopathy, and allograft renal dysfunction or rejection in patients with renal transplant [5–7], but only limited data are available about viral infection and chemotherapy. BK virus could be identified in 0.3 to 6% of blood samples of normal population. Pathogenesis of BK virus infections consists into the necrosis of urothelial cells associated to the presence of an inflammatory infiltrate. Diagnosis of BK virus infections is made by the detection of viral DNA in blood or urine samples or by the presence of DECOY-CELLS (epithelial cells with enlarged nuclei, deeply basophilic intranuclear ground-glass inclusions) in urine. Classical treatment in immunocompromised host consists in systemic antiviral agents, Ganciclovir or Cidofovir.

In our case, the viral infection was suspected before classical presentation as hemorrhagic cystitis. The presence of BK virus into urine samples allowed us to make the differential diagnosis between hemorrhagic cystitis due to Endoxan administration and viral infection. Because of absence of viremia, no specific treatment was performed; the symptoms regressed spontaneously after ten days. A new analysis of urine sample found the diminution of viral charge. There was no relapse of the disease in one year of followup.

We believe that in case of apparition of hemorrhagic cystitis in a context of recent immunosuppressive chemotherapy, viral research in urine and blood samples must be performed, and an antiviral treatment must be started only in case of viremia.
